# Structural correlates of formal thought disorder in schizophrenia: An ultra-high field multivariate morphometry study

**DOI:** 10.1016/j.schres.2015.07.022

**Published:** 2015-10

**Authors:** Lena Palaniyappan, Jenaid Mahmood, Vijender Balain, Olivier Mougin, Penny A. Gowland, Peter F. Liddle

**Affiliations:** a Translational Neuroimaging for Mental Health, Division of Psychiatry & Applied Psychology, Institute of Mental Health, University of Nottingham, UK; b Early Intervention in Psychosis, Nottinghamshire Healthcare NHS Trust, Nottingham, UK; c Penticton Regional Hospital, Penticton, British Columbia, Canada; d Sir Peter Mansfield Magnetic Resonance Centre, School of Physics, University of Nottingham, UK

**Keywords:** Disorganisation, Formal thought disorder, Morphometry, Grey matter volume, Insula, Cingulate cortex, Striatum

## Abstract

**Background:**

Persistent formal thought disorder (FTD) is one of the most characteristic features of schizophrenia. Several neuroimaging studies report spatially distinct neuroanatomical changes in association with FTD. Given that most studies so far have employed a univariate localisation approach that obscures the study of covarying interregional relationships, the present study focussed on the multivariate systemic pattern of anatomical changes that contribute to FTD.

**Methods:**

Speech samples from nineteen medicated clinically stable schizophrenia patients and 20 healthy controls were evaluated for subtle formal thought disorder. Ultra high-field (7 T) anatomical Magnetic Resonance Imaging scans were obtained from all subjects. Multivariate morphometric patterns were identified using an independent component approach (source based morphometry). Using multiple regression analysis, the morphometric patterns predicting positive and negative FTD scores were identified.

**Results:**

Morphometric variations in grey matter predicted a substantial portion of inter-individual variance in negative but not positive FTD. A pattern of concomitant striato-insular/precuneus reduction along with frontocingular grey matter increase had a significant association with negative FTD.

**Conclusions:**

These results suggest that concomitant increase and decrease in grey matter occur in association with persistent negative thought disorder in clinically stable individuals with schizophrenia.

## 1 Introduction

Formal thought disorder (FTD) is one of the defining features of schizophrenia. FTD is closely related to the constructs of disorganisation syndrome and hebephrenia and has been reported in 80–90% of individuals with acute psychosis in some samples (Andreasen, 1979; Harrow et al., 1986; Harrow and Marengo, 1986). In particular, persistence of FTD is considered as a core feature of the long-term course of schizophrenia (Harvey et al., 1984). In a follow-up study persistent FTD was observed in 64% of patients with schizophrenia, while only 33% patients with non-schizophreniform psychosis displayed such persistence 7.5 years after the first psychotic episode (Marengo and Harrow, 1997).

Structural basis of persistent thought disorder has been a matter of interest for the last 20 years, but continues to be unclear. A large majority of studies have focused on the superior temporal gyrus (STG) as a region-of-interest (ROI) while examining the structural basis of FTD. Several (Menon et al., 1995; Shenton et al., 1992; Subotnik et al., 2003; Weinstein et al., 2007) but not all (Flaum et al., 1995; Kim et al., 2003; Molina et al., 2003) of these studies have found an association between reduced volume of STG and the severity of FTD. Morphometric abnormalities in other ROIs such as the inferior frontal gyrus (Suga et al., 2010), supramarginal gyrus (Palaniyappan and Liddle, 2012), orbitofrontal cortex (Nakamura et al., 2008), cerebellum (Kühn et al., 2012), amygdala (Rajarethinam et al., 2001) and parahippocampal gyrus (Prasad et al., 2004) have also been related to disorganisation or FTD. Whitford et al. (2005) and Lui et al. (2009) undertook whole brain voxelwise studies comparing patients and controls, but restricted the study of disorganisation to ROIs that showed significant effect of diagnosis, thus precluding unbiased inference on the spatial distribution of structural correlates of FTD. A small number of whole brain studies have investigated the association between disorganisation/FTD and morphometric changes across the entire brain without prior anatomical assumptions. STG volume reductions were noted in some (Horn et al., 2009, 2010; Leube, 2009; Sans-Sansa et al., 2013) but not all (Chua et al., 1997; Rigucci et al., 2013) studies. Other regions showing FTD-related morphometric changes in the whole brain studies include the cerebellum (Leube, 2009; Rigucci et al., 2013), insula (Leube, 2009; Sans-Sansa et al., 2013), OFC (Horn et al., 2010; Sans-Sansa et al., 2013), anterior cingulate cortex (Horn et al., 2009; Sans-Sansa et al., 2013), hippocampal region (Chua et al., 1997), lingual gyrus (Horn et al., 2010), occipital lobe (Horn et al., 2010; Rigucci et al., 2013), precuneus (Horn et al., 2009), angular gyrus (Horn et al., 2009) and temporal pole (Horn et al., 2010). In summary, morphometric studies to date implicate distributed brain regions to be relevant to the pathophysiology of FTD, though no consistent reports have emerged.

Inconsistent observations of structural changes in relation to FTD could be attributed to various factors. Firstly, previous morphometric studies have adopted a univariate approach in seeking the structural basis of FTD. These studies assume that between subjects, regional variations in brain structure are spatially distinct, and do not take into account the covariance or interrelationship that exists among distributed regions. This issue is especially important in the investigation of schizophrenia, where structural changes affecting distributed ‘systems’ in the brain, rather than single regions, are suspected to underlie the complex clinical symptoms observed in patients. Secondly, the prominence of FTD varies with the clinical stage of the psychotic illness (Arndt et al., 1995; Russo et al., 2013). It is possible that only a small portion of the variance in such state-related symptom severity could be related to the structural variations in the brain (Mathalon and Ford, 2012). In contrast, morphometric changes might relate better to persistent, trait-like FTD seen in clinically stable subjects despite adequate treatment. Moreover, subtle aspects of FTD are often missed during the course of clinical interactions (De Bruin et al., 2007); an adequate assessment of FTD requires unstructured, freely generated speech samples (Johnston et al., 1986; Liddle et al., 2002a; Kircher et al., 2014). Finally, most structural MRI studies (except Sans-Sansa et al., 2013) have sought the neural correlates for overall severity of FTD as a single construct, though functional imaging studies indicate that the pathophysiology of positive FTD characterized by looseness and peculiar word, sentence or logic usage may differ from negative FTD characterized by poverty of speech and weakening of goal (Kircher et al., 2001, 2003; McGuire et al., 1998a,b).

A multivariate statistical approach called source-based morphometry (SBM) offers a novel means to study the patterns of morphometric variations in grey matter in relation to disease states (Xu et al., 2009). In this approach, the term ‘source’ refers to independent spatial components derived without any a-priori assumptions that have similar patterns of morphometric variability between subjects. Due to its multivariate nature, SBM reveals linked sub-systems or ‘macrocircuits’ in the brain that relate to a clinical phenotype (Caprihan et al., 2011). Several recent studies have utilized this approach to investigate clinical features (Kubera et al., 2013; Wolf et al., 2014) in psychotic disorders. In the present study, we aimed to identify spatially independent, SBM-derived grey matter components at a whole brain level that predict positive and negative FTD in clinically stable subjects with schizophrenia. To this end, we quantified FTD using freely generated speech sample. Further, we collected structural scans from an ultra high field 7-Tesla MRI scanner. 7 T MRI offers higher signal-to-noise resolution compared to lower field scans (Metcalf et al., 2010), and offers superior accuracy in discriminating structural changes seen in patients with schizophrenia (Iwabuchi et al., 2013). Given the inconsistency of previous structural studies, we made no a priori assumptions as to the brain regions implicated in FTD, but hypothesized the presence of structural alterations across distributed anatomical ‘subsystems’ in relation to the variations in severity of persistent FTD in clinically stable patients with schizophrenia.

## 2 Methods

### 2.1 Participants

The characteristics of the sample used in this study have been described previously (Iwabuchi et al., 2013). 20 patients and 21 healthy controls aged between 18–55 years were recruited, of which 19 patients and 20 controls had scans of adequate quality for morphometric analyses. Patients satisfied the diagnostic criteria for schizophrenia according to DSM-IV criteria assessed on the basis of a consensus procedure based on a review of case files, information from the psychiatrists providing direct clinical care and a structured clinical interview (Signs and Symptoms of Psychotic Illness, (Liddle et al., 2002b)) to assess clinical features. Patients were specifically recruited in a stable phase of illness (defined as a change of no more than 10 points in their Global Assessment of Function (GAF, DSM-IV) score, assessed six weeks prior and immediately prior to study participation). Patient recruitment was carried out in Nottinghamshire, from cases registered with generic community mental health teams, first episode teams or rehabilitation teams. The mean duration of illness was 7.7 years (SD = 8.3). Subjects with neurological disorders, current substance dependence, or IQ < 70 using Quick Test (Ammons and Ammons, 1962) were excluded. Healthy controls group-matched for age, gender and parental socioeconomic status were recruited from the local communities. Controls had no personal or family history of psychosis and were free of neuropsychiatric disorders as assessed by a research psychiatrist. The study was conducted in Nottinghamshire, UK with ethical permission obtained from the National Research Ethics Committee, Nottingham. All participants gave written informed consent and received monetary compensation for their time in accordance with the ethical approval.

### 2.2 MRI data acquisition

Scanning was performed on a 7 T Philips Achieva system with 32-channel receive coil. T1 weighted images were acquired using a 3D Magnetization Prepared-Turbo Field Echo (IR-TFE) with 0.6 mm isotropic resolution, 192 × 180 × 140 mm matrix, TR = 15 ms, TE = 5.6 ms, shot interval = 3 s, and flip angle 8°. An optimized inversion pulse (adiabatic pulse) was used at 7 T to reduce bias field inhomogeneity. One patient and one control were excluded due to significant movement artefacts. T1 weighted images were resliced (1 mm isotropic) and segmented into grey, white and CSF tissue using the SPM8 Diffeomorphic Anatomical Registration Through Exponentiated Lie algebra (DARTEL) algorithm (Ashburner, 2007) using a study-specific template. To correct for variation due to field inhomogeneity, the images were bias field corrected using 60 mm FWHM setting using SPM8 (Uwano et al., 2013). Further precautions taken to reduce bias field inhomogeneity are described in our previous work (Iwabuchi et al., 2013). Modulated grey matter images were normalized to MNI space using DARTEL's high-dimensional normalization protocol and smoothed using a 8 mm isotropic Gaussian kernel in line with recent SBM studies (Kubera et al., 2013; Wolf et al., 2014). Intracranial volume was calculated as a sum of the partial volumes derived from grey matter, white matter and CSF tissue images.

### 2.3 Assessment of thought disorder

Subjects were interviewed on the same day as the scan to assess seven features of formal thought disorder (poverty of speech, weakening of goal, perseveration, looseness, peculiar word usage, peculiar sentence usage and peculiar logic) in line with the validated procedure for administering Thought Language Index (Liddle et al., 2002a). To generate free speech samples, 3 pictures from Thematic Apperception Test (Murray, 1943) were used as in the study by Sommer et al. (2010). Speech samples were audio recorded by two research psychiatrists (LP and VB) and transcribed and rated by a single author (JM) blind to the diagnostic status, symptom burden of the subjects and neuroimaging findings. During the training phase, several meetings were organized among the authors to develop consensus on how to interpret and score FTD using previously collected speech samples. Further, we used the examples from the original author (PFL) for reference. In keeping with the original description of the scale, the summed scores of looseness, peculiar word, peculiar sentence and peculiar logic were classified as positive FTD (disorganised thinking) and the summed scores of perseveration, poverty of speech and weakening of goal were termed as negative FTD (impoverished thought). The inclusion of perseveration with negative FTD has been supported by some (Andreasen and Grove, 1986) but not all factor analyses (Liddle et al., 2002a). We repeated our primary analysis (reported below) after excluding perseveration scores from negative FTD. This did not affect the overall results (Supplementary material).

## 3 Source based morphometry

SBM analysis was carried out using Group ICA Toolbox as per the standard descriptions provided by the authors (Xu et al., 2009). For this procedure, each grey matter image was converted to a one-dimensional vector of voxel-specific values and arrayed into a 39-row subjects-by-voxels matrix. The number of independent components was estimated using minimum description length criteria (Li et al., 2007). The subjects-by-voxels matrix was decomposed into a mixing matrix (subjects-by-components) representing loading parameters that quantify the contribution of each subject to the group for a given component and source matrix (components-by-voxels) representing the spatially independent ‘sub-systems’ defined on the basis of morphometric covariance within the group (see the Supplementary material for further details). We employed a bootstrapping algorithm (20 iterations) to increase the stability of the estimated components (Himberg et al., 2004). Further second-level statistical analysis was carried out on the loading parameters. To visualize the spatial components, the source matrix was recomposed to statistical maps in 3 dimensional MNI space with coefficients expressed in standard deviation units (z-maps). The anatomical descriptions of these maps were obtained using Talairach coordinates utility in the GIFT toolbox.

The grey matter intensity in each voxel in each subject (after removal of the group mean) is the sum of 8 product values obtained from multiplying the voxel loading for each component with its coefficient for that subject. As a result, the sign of the loading coefficients of a component in a subject do not directly provide the direction of change in absolute grey matter intensity in a region (Caprihan et al., 2011). To address this sign ambiguity and to directly interpret the direction of morphometric changes within the spatial components, we derived the mean grey matter volume from all voxels with z > 2 within a component for each subject, and adjusted the signs of the ICA maps so that the correlation of loading coefficients with grey matter volume is positive within each map. This procedure, in line with Caprihan et al. (2011), allowed us to interpret whether an overall increase or decrease in component-specific grey matter accounted for a higher burden of FTD in an individual (Supplementary material).

## 4 Statistical analysis

All statistical tests were carried out using SPSS version 21.0 (IBM Corp., Armonk, NY). Clinical and demographic variables were compared between patients and controls using t tests (or Mann–Whitney U tests for non-normal data) and chi-square tests for proportions. A patient vs. controls comparison on the 8 spatial components was performed using multivariate analysis of variance (MANOVA) followed by Bonferroni corrected univariate ANOVAs for each component. Age, gender and intracranial volume were used as covariates for this analysis as these variables could affect the regional grey matter distributions. In the patient group, multiple regression analyses were conducted separately to predict positive and negative FTD using all of the 8 ICA parameters, entered simultaneously into the regression models after excluding multicollinearity using a tolerance threshold of 0.1. The relationship between current antipsychotic dose and the grey matter components was studied by relating the loading parameters with Defined Daily Dose equivalents of antipsychotics (WHO Collaborating Centre for Drug Statistics and Methodology, 2003) using Spearman's correlation. We did not have data on cumulative antipsychotic exposure for this sample.

## 5 Results

Clinical and demographic features of the sample are presented in Table 1. The two groups were well matched for age, gender and parental socioeconomic status. Positive FTD was seen only in patients, but negative FTD was noted in both patients and controls, with higher degree of severity in patients.

**Fig. 1:**
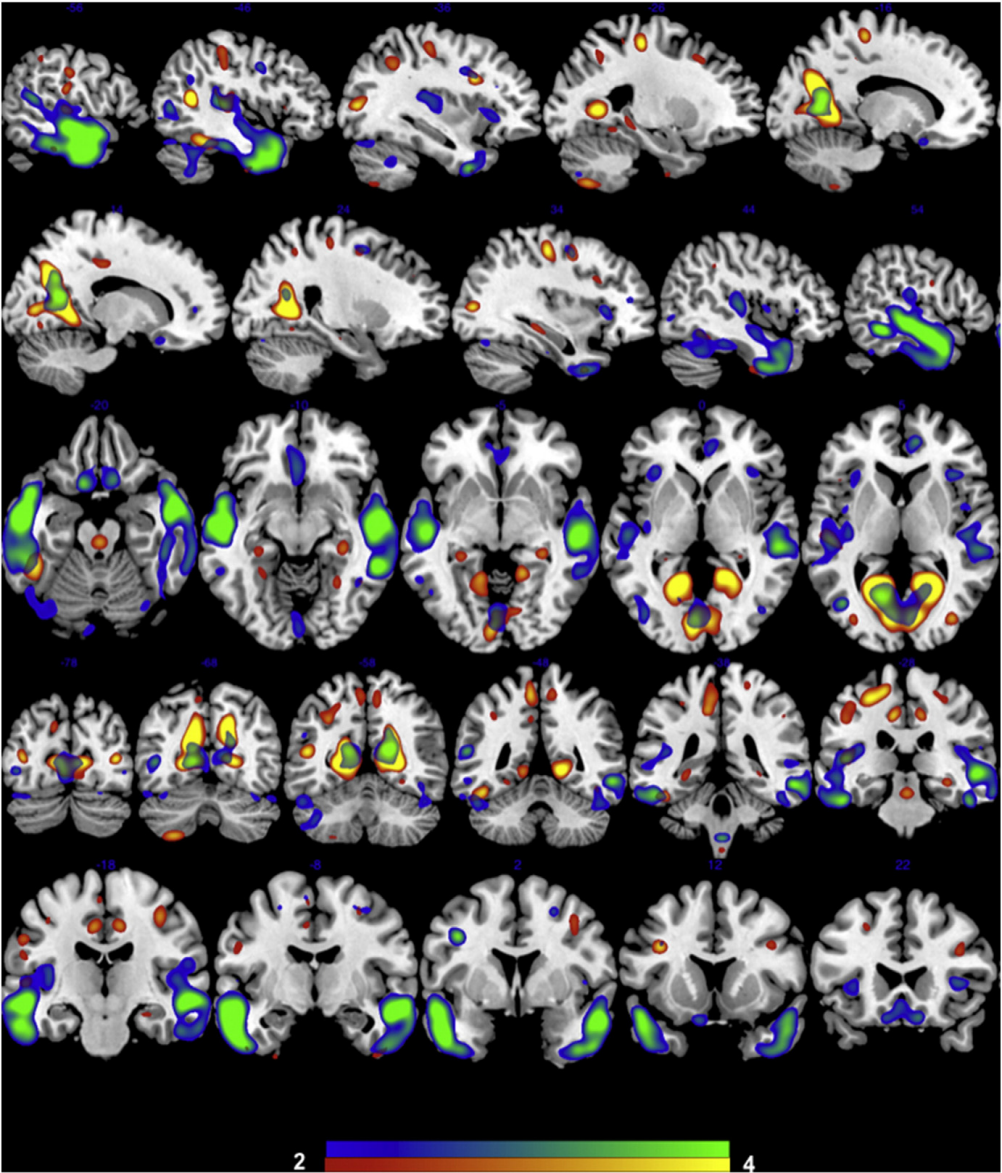
Independent spatial components with reduced grey matter in patients compared to controls. IC2 is displayed in yellow-red; IC6 is displayed in blue-green. Slices selected for the best display of the 2 components, overlaid on a template structural image using MRIcron software. The components are thresholded at z > 2.

The minimum description length criteria revealed 8 independent spatial components with covarying grey matter patterns in the SBM analysis. The anatomical description of the regions included in each component is presented in Table 2. MANOVA revealed a significant group difference between patients and controls in the loading coefficients [*F*(8, 27) = 2.72, *p* = 0.024]. The effect of diagnosis was most pronounced for IC2 [*F*(1, 34) = 11.9, *p* = 0.008; coefficient mean(SD) in controls = 0.44(0.18), patients = − 0.46(0.19)] and IC6 [*F*(1, 34) = 9.7, *p* = 0.03; coefficient mean(SD) in controls = 0.23(0.11), patients = − 0.24(0.11)]. No other significant between-group differences were noted. IC2 included the precuneus, posterior cingulate, superior temporal, lingual and fusiform gyrus, middle occipital, precentral and paracentral regions and middle frontal gyrus. IC6 included the superior, middle and inferior temporal gyrus, precuneus and posterior cingulate regions, medial and inferior frontal regions. The spatial distribution of these two components is presented in Fig. 1. In both IC2 and IC6, controls had a higher volume of grey matter than patients.

**Fig. 2:**
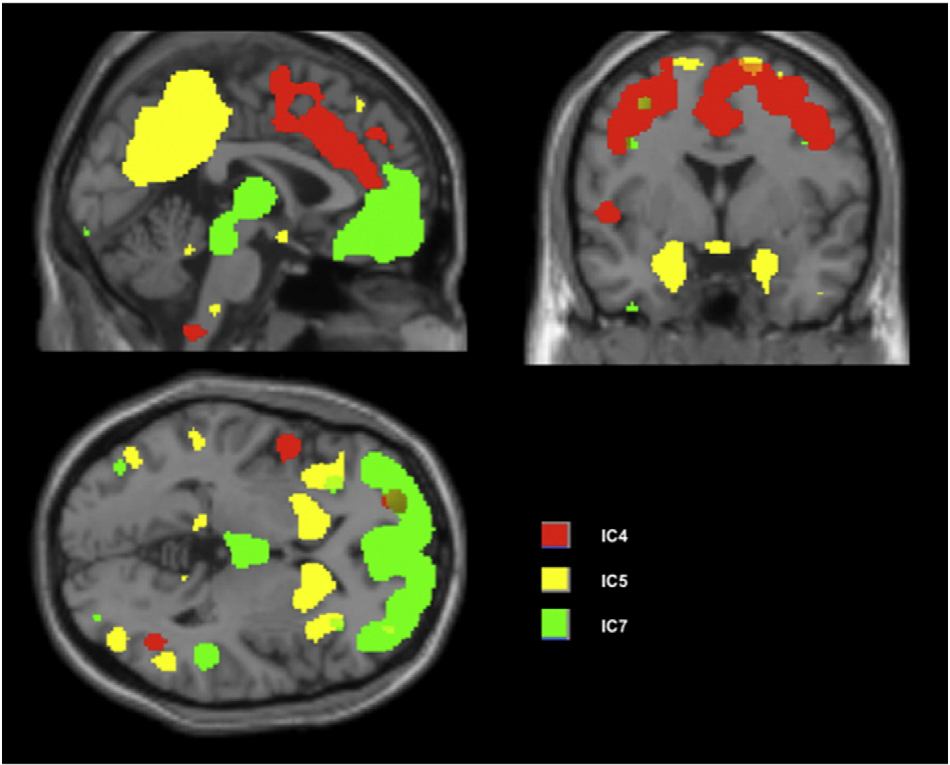
Independent spatial components (ICs) related to negative FTD in patients. Loading coefficients of IC4 and IC7 increased while IC5 decreased with higher severity of negative FTD. Binary masks of the components thresholded at z > 2, overlaid on single subject anatomical image provided with xjView software (www.alivelearn.net/xjview8). See Table 2 for anatomical description of the ICs.

The regression models were significant for negative FTD (F = 2.65, *p* = 0.026) with the loading coefficients of IC4, IC5 and IC7 (Fig. 2) significantly predicting negative FTD in patients. These three variables explained most of the variance (adjusted R^2^ = 67%) in the negative FTD scores. Higher coefficient values in IC4 (dorsal anterior cingulate, superior, middle and medial frontal regions) and IC7 (superior, inferior, middle and medial frontal), and lower values in IC5 (striatum, insula, superior temporal, posterior cingulate and precuneus) were associated with negative FTD (Table 3). The multiple regression models were not significant for predicting positive FTD in patients (F = 2.65, *p* = 0.026) or the clinical syndrome scores (reality distortion, psychomotor poverty and disorganisation) obtained from the SSPI interview (further details in the Supplementary material). In controls, the morphometric variations in the 8 spatial components did not predict the negative FTD (F = 1.22, *p* = 0.38). Daily doses of antipsychotics (current exposure) were not related to the grey matter variations in any of the SBM derived components (all *p* > 0.4) (Supplementary Material).

## 6 Discussion

To our knowledge, this is the first study employing a multivariate morphometric procedure to study thought disorder in schizophrenia. In this study, we have shown that (1) clinically stable and medicated individuals with schizophrenia exhibit FTD that is quantifiable by analyzing speech samples, (2) a significant proportion of the variance in *negative* FTD is explained by morphometric variations in distributed brain regions (especially frontotemporal cortex, striatum, insula, anterior and posterior cingulate cortex and precuneus) and (3) both increased and decreased grey matter volumes are noted in association with FTD.

With respect to the regression analysis, we noted that a reduction in grey matter volume involving the striatum, insula, precuneus and lateral temporal regions predicted higher burden of negative FTD, in the presence of an increase in grey matter volume involving the cingulate and lateral prefrontal regions. It is important to note that our results point to *a pattern* of concomitant changes (both increases and decreases in GMV), rather than specific regional changes, that best explain the variance in negative FTD seen among clinically stable patients with schizophrenia. While not directly comparable, these observations are partly consistent with previous whole brain structural and functional studies implicating the insula (Leube, 2009; Liddle et al., 1992), striatum (Ebmeier et al., 1993; Liddle et al., 1992) precuneus (Horn et al., 2009) and temporal cortex (Horn et al., 2009, 2010), though these studies did not distinguish positive and negative FTD. To our knowledge only one previous study (Sans-Sansa et al., 2013) has examined the structural correlates of negative FTD. This study used Andreasen's Thought, Language and Communication scale (Andreasen and Grove, 1986) and employed voxelwise mass univariate approach. In Sans-Sansa et al.'s study, poverty of content of speech was related to grey matter reductions in the orbitofrontal and insular cortex, while poverty of speech was not quantified. In addition, positive FTD was associated with reduced grey matter volume in the superior temporal cortex and inferior frontal cortex. These results are partly consistent with reduced insular volume predicting severe negative FTD in our sample. Nevertheless, we did not observe any structural basis for the positive FTD. While we recruited clinically stable patients (not enriched for the presence of thought disorder) to study otherwise subtle FTD, Sans-Sansa et al. recruited hospitalized patients with chronic illness selected for the presence of pronounced FTD (enriched sample). It is likely that such pronounced positive FTD in chronic patients is associated with greater structural abnormality than the subtler positive thought disorders in the outpatient sample in the current study. Further, the variance of positive FTD (σ^2^ = 0.4) was limited compared to that of negative FTD (σ^2^ = 0.8) in our patient sample, thus restricting our ability to detect a relationship with brain structure.

Our observations suggest that *increased* grey matter volume in the prefrontal regions and dorsal anterior cingulate predicted more severe negative FTD in patients, in the presence of *concomitant GM reduction* in the bilateral insula, precuneus and striatum. Insofar as SBM reveals the ‘macrocircuit’ patterns in grey matter structure, this observation suggests that a structural imbalance between the insula/striatal macrocircuit on one hand and the frontocingular system on the other could contribute to negative FTD. Given the predominance of volumetric reduction in schizophrenia, the observation of a structural pattern involving an increase in GMV in certain regions in relation to thought disorder appears counterintuitive initially. But an increase in grey matter concentration in relation to thought disorder (albeit positive FTD) has been previously demonstrated in schizophrenia (Chua et al., 1997). An increase in frontocingular volume could also be a secondary, probably an inefficient and inadequate compensatory response to the presence of thought disorder. Alternatively, patients with persistent FTD might have had a higher lifetime exposure of antipsychotic treatment that could have confounded the volumetric changes (Stip et al., 2009). But at present, longitudinal observational evidence in this regard suggests that antipsychotic prescription is associated with reduced, rather than increased frontal volume (Ho et al., 2011) (also see the Supplementary material). The presence of increased prefrontal grey matter in patients with persistent thought disorder is a novel observation arising from our multivariate approach; this suggests that the structural correlates of FTD involve both increases and decreases in grey matter volume.

With respect to the group contrast, we noted a significant reduction in the grey matter volume affecting the bilateral temporal lobe, fusiform and lingual gyrus, precuneus/posterior cingulate cortex and several distributed regions in the frontal cortex and the insula. These findings replicate the two previous multivariate morphometric observations that employed SBM to study grey matter changes in schizophrenia. Xu et al. (2009) reported a significant bilateral temporal grey matter reduction along with and distributed reductions in the lateral frontal, insular, lingual gyrus, and precuneus regions. Kaspárek et al. (2010) studied a first episode sample and reported bilateral temporal and reductions along with distributed changes in other regions. The presence of grey matter reduction affecting the lateral temporal macrocircuit appears to be a consistent feature across studies in schizophrenia.

Our study has several strengths including the use of ultra high-field imaging, employing an objective measure of FTD using freely generated speech samples, and the use of multivariate morphometric technique to study a brain–symptom relationship. Several limitations must also be borne in mind while interpreting the results of this study. The size of our sample was limited, though comparable to several previous whole brain studies of FTD (Horn et al., 2009, 2010). We recruited a medicated, clinically stable sample; while this might have contributed to a reduction in the variance of symptom scores, this offered an opportunity to study the persistent, stable, trait-like aspect of negative FTD. Further the confounding effects of antipsychotics cannot be ruled out. Though we studied the relationship between currently prescribed dose and the brain structure, we lacked longitudinal data on antipsychotic exposure.

In summary, using a multivariate morphometric analysis, we have demonstrated both increases and decreases in grey matter in association with persistent negative thought disorder in clinically stable individuals with schizophrenia. Our results suggest that several sub-systems (or macrocircuits) are likely to be involved in the pathophysiology of FTD; further longitudinal studies with multiple assessments of FTD are required to clarify whether some of these represent a compensatory rather than primary change.

## Conflicts of interest

L Palaniyappan received a travel fellowship sponsored by Eli Lilly in 2011, and support in kind from Magstim Company Ltd for a conference presentation in 2014. In the past five years, P F Liddle has received honoraria for academic presentations from Janssen-Cilag and Bristol Myers Squibb; and has taken part in advisory panels for Bristol Myers Squibb. All other authors declare no conflict of interest.

## Contributors

Data reported here was collected as a part of doctoral study of LP supervised by PFL. The hypothesis tested in this study was conceived by LP who supervised JM to undertake the statistical analysis. All the authors have participated and have made substantial contributions to this paper: LP & VB: recruitment, clinical data collection, analysis, interpretations of data and preparing the manuscript. OM: imaging data collection, analysis, interpretations of data and preparing the manuscript. JM: clinical data collection, analysis, interpretations of data and preparing the manuscript. PAG: supervision of the study, interpreting the data and preparing the manuscript. PFL: design, conception and supervision of the study, interpreting the data and preparing the manuscript. All authors have read and approved the final version of the article.

## Role of funding sources

We are grateful for the support received from the Medical Research Council (G0601442) that helped us to develop and optimize the image acquisition protocol. L Palaniyappan was supported by a research training fellowship from the Wellcome Trust during the period of this work (WT096002/Z/11). This work was funded by an internal grant from the School of Community Health Sciences, University of Nottingham to L Palaniyappan.

## Figures and Tables

**Table 1:** Clinical and demographic features.

Features	Patients (N = 19) Mean(SD)	Controls (N = 20) Mean(SD)	Statistic
Gender (male/female)	14/5	15/5	χ^2^ = 0.93
Handedness (right/left)	17/2	18/2	χ^2^ = 0.45
Age	33.2(9.8)	32(8.2)	t = 0.44
Parental NS-SEC	2.6(1.7)	2.5(1.6)	z = 0.16
SSPI total score	11.1(10)	0.6(0.8)	z = 5.0⁎
Reality distortion	2.4(2.9)	0(0)	z = 3.9⁎
Disorganisation	0.2(0.7)	0.1(0.3)	z = 3.0⁎
Psychomotor poverty	2.3(3.7)	0(0)	z = 2.9⁎
Illness duration (years)	7.7(8.3)	–	
DDD of antipsychotics	0.8(0.7)	–	
GAF score	47.3(10.7)	88.1(7.4)	t = − 13.9⁎
Impoverished thinking (negative FTD)	0.486(0.89)	0.042(0.10)	z = 2.16⁎^a^
Disorganised thinking (positive FTD)	0.556(0.67)	0(0)	z = 4.31⁎^a^
Intracranial volume (ml)	1558.6(169.1)	1593.3(146.8)	t = − 0.68

SD: Standard deviation. NS-SEC: National Statistics Socio-economic Classification. SSPI: Signs and Symptoms of Psychotic Illness. DDD: Defined Daily Dose. GAF: Global Assessment of Functioning.

a 2 patients and 1 control excluded for missing values due to poor quality of audio transcripts.

⁎ Significantly different between the two groups at *p* < 0.05.

**Table 2:** Anatomical description of the independent components.

Anatomical regions	Max z value (Talairach coordinates x,y,z) for left/right hemispheres.
*Component IC1*	
Precuneus	4.1 (− 39, − 65, 35)/8.8 (25, − 62, 35)
Superior temporal gyrus	4.5 (− 46, − 57, 28)/6.6 (46, − 50, 15)
Middle temporal gyrus	na/6.3 (50, − 43, 5)
Supramarginal gyrus	6.2 (− 55, − 39, 36)/4.9 (55, − 46, 35)
Postcentral gyrus	5.0 (− 56, − 14, 33)/6.1 (52, − 20, 34)
Inferior parietal lobule	4.7 (− 59, − 40, 24)/5.6 (59, − 29, 32)
Middle frontal gyrus	4.9 (− 36, 26, 32)/na
Angular gyrus	na/4.9 (43, − 61, 33)
Precentral gyrus	4.6 (− 46, − 20, 37)/4.1 (56, − 1, 15)
Inferior frontal gyrus	3.6 (− 40, 42, 2)/na

*Component IC2*	
Precuneus	7.7 (− 16, − 60, 31)/8.1 (15, − 60, 20)
Posterior cingulate	7.2 (− 21, − 56, 8)/7.4 (18, − 56, 7)
Cuneus	4.7 (− 9, − 78, 8)/6.7 (12, − 66, 30)
Superior temporal gyrus	na/5.6 (45, − 52, 15)
Lingual gyrus	5.2 (− 15, − 48, 2)/5.0 (6, − 83, 4)
Precentral gyrus	4.1 (− 33, − 19, 49)/4.6 (28, − 22, 52)
Middle occipital gyrus	4.3 (− 37, − 77, 11)/3.8 (36, − 77, 12)
Middle frontal gyrus	3.5 (− 33, − 1, 46)/4.1 (36, 11, 26)
Paracentral lobule	na/4.0 (9, − 38, 54)
Fusiform gyrus	na/3.9 (45, − 43, − 14)
Cingulate gyrus	3.7 (− 10, − 20, 40)/3.7 (9, − 21, 40)

*Component IC3*	
Inferior semi-lunar lobule	6.7 (− 28, − 71, − 42)/5.9 (15, − 69, − 41)
Nodule	4.9 (− 3, − 52, − 30)/na
Declive	4.5 (− 34, − 59, − 22)/4.3 (30, − 62, − 21)
Uvula	4.3 (− 9, − 69, − 32)/4.0 (7, − 66, − 33)
Cerebellar tonsil	3.9 (− 40, − 50, − 42)/na
Lingual gyrus	na/3.9 (13, − 72, − 4)
Culmen	3.7 (− 36, − 48, − 25)/3.8 (36, − 52, − 24)
Pyramis	3.8 (− 25, − 74, − 27)/na

*Component IC4*	
Middle frontal gyrus	6.7 (− 27, − 8, 53)/7.4 (15, − 7, 61)
Superior frontal gyrus	3.8 (− 27, − 8, 64)/5.2 (12, 19, 54)
Inferior parietal lobule	5.2 (− 39, − 46, 40)/na
Inferior frontal gyrus	4.0 (− 48, 6, 29)/4.3 (46, 4, 30)
Anterior cingulate	4.0 (− 7, 30, 23)/4.1 (6, 29, 23)
Cingulate gyrus	3.7 (− 7, 22, 35)/na
Medial frontal gyrus	3.7 (− 7, − 10, 60)/na

*Component IC5*	
Lentiform nucleus	7.5 (− 19, 11, − 6)/7.0 (18, 11, − 6)
Cingulate gyrus	6.3 (− 7, − 42, 35)/4.8 (4, − 37, 39)
Parahippocampal gyrus	na/5.2 (22, − 10, − 17)
Caudate	4.8 (− 12, 18, 1)/3.7 (10, 15, 2)
Middle temporal gyrus	3.6 (− 43, − 61, 10)/4.5 (42, − 64, 11)
Precuneus	4.4 (− 4, − 50, 43)/4.2 (7, − 46, 33)
Superior frontal gyrus	4.4 (− 15, 16, 57)/4.0 (16, 48, 28)
Precentral gyrus	na/4.3 (37, 13, 34)
Superior temporal gyrus	4.2 (− 43, − 51, 25)/na
Middle occipital gyrus	4.1 (− 40, − 76, 1)/na
Paracentral lobule	na/4.0 (4, − 37, 50)
Posterior cingulate	3.8 (− 1, − 60, 22)/na
Insula	na/3.8 (33, 18, 6)
Inferior temporal gyrus	3.7 (− 56, − 17, − 16)/na
Postcentral gyrus	3.7 (− 7, − 48, 65)/na
Inferior occipital gyrus	na/3.5 (43, − 75, − 5)

*Component IC6*	
Middle temporal gyrus	5.6 (− 52, − 7, − 14)/5.9 (52, − 11, − 12)
Fusiform gyrus	3.7 (− 61, − 13, − 23)/5.4 (50, − 3, − 23)
Posterior cingulate	na/4.3 (15, − 61, 10)
Superior temporal gyrus	3.6 (− 58, − 29, 3)/4.2 (49, 11, − 17)
Inferior frontal gyrus	na/4.1 (40, 2, 33)
Inferior temporal gyrus	3.8 (− 49, − 2, − 33)/4.1 (48, − 5, − 33)
Precuneus	3.9 (− 16, − 58, 29)/na
Medial frontal gyrus	na/3.7 (10, 15, − 17)
Middle occipital gyrus	na/3.5 (42, − 67, 9)

*Component IC7*	
Superior frontal gyrus	6.8 (− 16, 64, − 7)/7.6 (24, 56, − 8)
Inferior frontal gyrus	3.8 (− 43, 38, 4)/7.6 (37, 42, 1)
Middle frontal gyrus	7.0 (− 27, 56, − 7)/4.9 (36, 52, − 5)
Medial frontal gyrus	6.1 (− 10, 64, 2)/5.2 (7, 61, 0)
Precuneus	na/5.4 (25, − 65, 35)
Superior parietal lobule	5.3 (− 28, − 55, 43)/na
Inferior parietal lobule	na/5.0 (49, − 40, 24)
Postcentral gyrus	na/4.3 (43, − 26, 37)
Anterior cingulate	4.0 (− 9, 47, − 1)/na
Middle temporal gyrus	3.8 (− 49, − 36, 5)/na

*Component IC8*	
Middle occipital gyrus	8.2 (− 37, − 80, 12)/7.2 (24, − 91, 9)
Cuneus	6.7 (− 12, − 89, 18)/6.3 (7, − 91, 14)
Middle temporal gyrus	4.5 (− 45, − 72, 17)/4.6 (43, − 72, 19)
Precuneus	4.2 (− 18, − 72, 40)/na
Inferior occipital gyrus	4.1 (− 27, − 91, − 4)/4.1 (36, − 82, − 5)
Superior occipital gyrus	na/3.5 (36, − 77, 27)

The anatomical regions within each component are summarized after thresholding the z-maps at z > 3.5. To avoid reduplication, only regions with positive contribution to the covariance are listed. na: no effect with z > 3.5.

**Table 3:** Multiple regression analysis.

Independent variables	Dependent variables	
Negative FTD F = 4.86(0.015) β(*p* value)	Positive FTD F = 0.50(0.82) β(*p* value)
IC1	− 0.18(0.39)	− 0.29(0.48)
IC2	− 0.14(0.66)	− 0.37(0.56)
IC3	0.13(0.73)	− 0.38(0.60)
IC4	**0.95(0.006)**	0.17(0.75)
IC5	**− 1.16(0.03)**	− 0.04(0.97)
IC6	0.20(0.61)	− 0.01(0.99)
IC7	**0.85(0.007)**	− 0.09(0.84)
IC8	− 0.15(0.56)	− 0.36(0.47)

Note: Values in bold letters are statistically significant. IC: Independent component. FTD: Formal thought disorder. SSPI: Signs and Symptoms of Psychotic Illness.

## Appendix A Supplementary data

Supplementary Materials with a visual summary of Source Based Morphometry and results of additional analyses.
