# Supplementary material for: Structural correlates of formal thought disorder in schizophrenia: An ultra-high field multivariate morphometry study
Source: Schizophr Res. 2015 Oct;168(1-2):305–12. doi: 10.1016/j.schres.2015.07.022 (PMC4604249; doi:10.1016/j.schres.2015.07.022)
Supplement: Supplementary file 1 — Supplementary Materials with a visual summary of Source Based Morphometry and results of additional analyses. [file mmc1.docx]

**Supplementary Materials**

**Source Based Morphometry**

Independent component analysis in SBM is based on the voxel-by-voxel covariance in the morphometric measure (grey matter volume) observed in a population. The figure below (based on Xu et al. 2009) summarizes the process of deriving ‘sources’ or components from grey matter images.

**Pearson’s correlation between sign corrected loading coefficients and mean grey matter volume of the IC maps thresholded at z>2.**

| **Components** | **Correlation coefficient r (all p<0.001)** |
| --- | --- |
| **IC1** | 0.886 |
| **IC2** | 0.669 |
| **IC3** | 0.980 |
| **IC4** | 0.881 |
| **IC5** | 0.973 |
| **IC6** | 0.975 |
| **IC7** | 0.941 |
| **IC8** | 0.887 |

**Multiple regression models predicting SSPI scores in patients and negative FTD in healthy controls**

| **Independent variables** | **Dependent variables**  β (p value) | | | |
| --- | --- | --- | --- | --- |
|  | SSPI Reality Distortion  F=0.47 (0.85) | SSPI Psychomotor Poverty  F=0.4(0.89) | SSPI Disorganisation  F=1.7(0.23) | Negative FTD in controls  F=1.22(0.38) |
| **IC1** | -0.26(0.5) | -0.09(0.81) | -0.26(0.39) | 0.18(0.64) |
| **IC2** | -0.06(0.91) | 0.60(0.31) | 0.79(0.09) | -0.21(0.49) |
| **IC3** | 0.10(0.89) | -0.02(0.98) | 0.28(0.61) | -0.43(0.49) |
| **IC4** | 0.13(0.77) | .038(0.93) | 0.23(0.51) | -0.89(0.23) |
| **IC5** | -0.52(0.44) | -0.48(0.48) | -1.25(0.03) | -0.01(0.97) |
| **IC6** | -0.14(0.83) | 0.57(0.41) | 0.87(0.11) | -0.30 (0.43) |
| **IC7** | 0.55(0.25) | 0.20(0.68) | 0.75(0.06) | 0.86(0.23) |
| **IC8** | -0.15(0.76) | -0.18(0.72) | -0.09(0.80) | 0.34(0.50) |

SSPI: Signs and Symptoms in Psychotic Illness IC: Independent components from the Source Based Morphometry analysis FTD: Formal Thought Disorder

**Excluding perseveration from negative FTD scores**

The inclusion of perseveration in negative FTD is controversial. Our observations suggest that when the grey matter volume represented by the loading coefficients of IC4,5 and 7 explained 67% of the variance in negative FTD when perseveration was included (F=12.6, adjusted R^2^=0.67, p<0.001). In this model, all three components significantly predicted the negative FTD scores [IC4 (β =0.92,t=4.2,p=0.001), IC5 (β=-0.85,t=-3.7,p=0.003) and IC7 (β =0.62,t=3.4,p=0.004)]. When perseveration was not included in the measurement of negative FTD, the multiple regression model continued to be significant with the three components explaining 63% of the total variance (F=10.8, adjusted R^2^=0.63, p=0.001). All three components continued to relate significantly to the negative FTD scores [IC4 (β=0.86,t=3.7,p=0.002), IC5 (β =-0.78,t=-3.2,p=0.007) and IC7 (β =0.62,t=3.2,p=0.006)].

**Spearman’s correlation between loading coefficient and Defined Daily Dose equivalents of antipsychotics**

| **Components** | **Spearman’s correlation coefficient r (p)** |
| --- | --- |
| **IC1** | -0.12(0.64) |
| **IC2** | -0.11(0.65) |
| **IC3** | 0.19(0.45) |
| **IC4** | -0.12(0.62) |
| **IC5** | 0.03(0.90) |
| **IC6** | 0.18(0.46) |
| **IC7** | -0.06(0.80) |
| **IC8** | 0.18(0.46) |
